# Extracorporeal Shock Wave Therapy Combined With Platelet-Rich Plasma Injection to Treat the Nonunion of a Stress Fracture of the Proximal Phalanx of the Great Toe: A Case Report

**DOI:** 10.7759/cureus.55877

**Published:** 2024-03-10

**Authors:** Toru Omodani

**Affiliations:** 1 Orthopaedics, Tokyo Advanced Orthopaedics, Tokyo, JPN

**Keywords:** proximal phalanx of the great toe, stress fracture, nonunion, platelet-rich plasma, extracorporeal shock wave therapy

## Abstract

A 15-year-old female short-distance track and field athlete started to experience pain in her left great toe while competing. One month after the onset of symptoms, she was diagnosed with a stress fracture of the proximal phalanx of the great toe. Despite three months of conservative treatment, no bone healing was observed, resulting in a nonunion. To promote healing of the fractured area, a treatment method involving the combination of extracorporeal shock wave therapy (ESWT) with platelet-rich plasma (PRP) injection was pursued. Complete bone healing was achieved six weeks after the start of the treatment, enabling the patient to fully return to her sport. Based on our findings, the combined use of ESWT and PRP injections, both beneficial for bone healing, is a potentially effective treatment for nonunion of the stress fracture of the proximal phalanx of the great toe.

## Introduction

Stress fractures of the proximal phalanx of the great toe are one of the overuse injuries occurring in the lower limb [1]. Traction force from the abductor hallucis muscle to the proximal phalanx of the great toe is implicated in its onset, and fractures typically occur on the medial and plantar sides [2]. It is considered a relatively rare condition, with only a few case reports available in the literature [3]. Conservative therapy, mainly involving rest from sports activities and resting the affected area, aiming for bone union, is commonly chosen first in these patients. If conservative therapy proves to be ineffective, or when faster bone union and recovery are desired, surgery may be considered. Munemoto et al. have reported two cases returning to competition in 8.5 weeks after undergoing conservative treatment [4]. Yokoe et al. reported that out of six cases treated with conservative therapy, three returned to sports within three months, while the other three cases experienced delayed union and ultimately required surgery [5]. As such, while it is possible to enable athletes to return to competition through conservative treatment, it may take some time, and in some cases, the delayed union may necessitate surgery.

To date, there have been no reports of conservative treatment combining extracorporeal shock wave therapy (ESWT) and platelet-rich plasma (PRP) injections to promote the healing of stress fractures of the proximal phalanx of the great toe. We present a case of a delayed union in a stress fracture of the proximal phalanx of the great toe that was treated with a combination of ESWT and PRP injection.

## Case presentation

A 15-year-old female, a short-distance track athlete, began experiencing pain in her left great toe during competition. One month after the onset of symptoms, she consulted a local clinic, where she was diagnosed with a stress fracture of the left proximal phalanx of the great toe based on radiograph and CT findings. After a month of rest, she gradually resumed training, but the pain during competitions did not improve. She once again halted her athletic activities, but the fracture did not heal. Three months after her initial consultation, she was referred to our hospital. She had no history of drug use or family history, and her menstrual cycle was normal. A radiograph confirmed that the stress fracture of the proximal phalanx of the great toe had progressed to a nonunion. Tenderness was observed at the fracture site, and pain was induced when stepping onto the forefoot. She expressed a strong desire to compete in an upcoming event scheduled seven weeks later. Intending to promote fracture healing, we decided on a treatment plan that combined ESWT and PRP injections.

**Figure 1:**
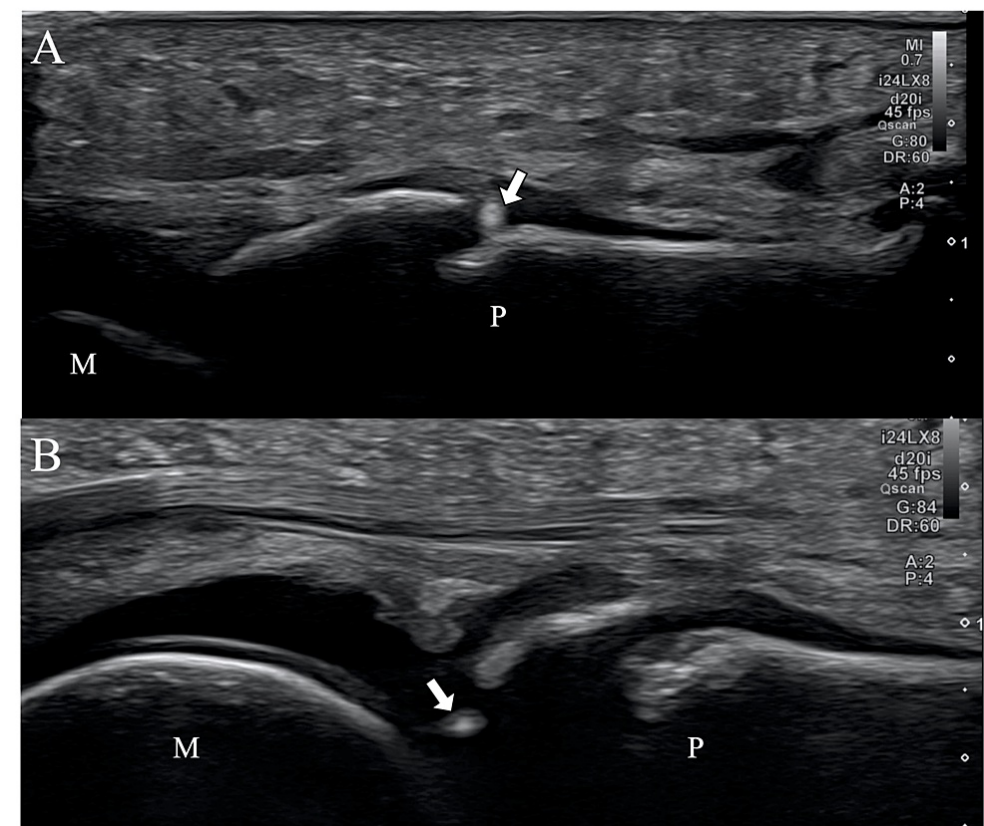
Image findings of ultrasound-guided PRP injection A: PRP was injected by inserting the needle tip into the fracture site of the proximal phalanx of the great toe. B: PRP was injected by inserting the needle tip into the metatarsophalangeal joint of the great toe. The arrow indicates the needle tip PRP: platelet-rich plasma; M: first metatarsal; P: proximal phalanx of the great toe

At the outset, we prepared PRP using the ACP Double Syringe System (Arthrex, Naples, FL). We collected 15 ml of venous blood from the anterior aspect of the patient's elbow and centrifuged it at 1500 rotations per minute for five minutes. The PRP separated in the upper layer was drawn off, yielding 4 ml of leucocyte-poor PRP. We observed the fracture site of the patient's great toe on the medial and plantar sides by placing a transducer longitudinally to the foot using an ultrasound machine (Aplio i700, Canon, Tokyo, Japan). Under ultrasound guidance, we inserted a 22-gauge needle subperiosteally at the fracture site and directly injected 2 ml of PRP. We also inserted a needle into the metatarsophalangeal joint and injected 2 ml of PRP into the joint (Figure 1). Next, we applied focused ESWT to the fracture site. Using the Duolith SD 1 (Storz Medical AG, Tägerwilen, Switzerland), we administered 2,500 pulses at an energy of 0.25 mJ/mm^2^. ESWT was performed every two weeks.

**Figure 2:**
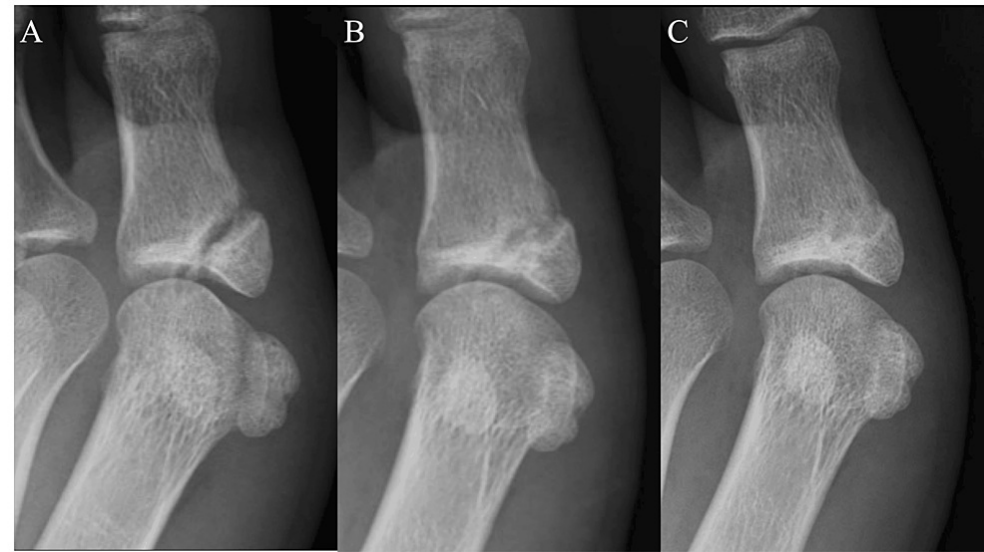
Radiographic findings of the left proximal phalanx of the great toe A: At the start of ESWT and PRP injection. B: Four weeks after treatment. C: Six weeks after treatment ESWT: extracorporeal shock wave therapy; PRP: platelet-rich plasma

Four weeks after the start of the treatment, good bone formation was observed on a radiograph. At this point, she gradually resumed training. Two weeks later, the radiograph confirmed complete bone union (Figure 2). There was no pain during training, and she was allowed to practice without restrictions. ESWT was concluded after a total of four sessions. Seven weeks after the start of treatment, the patient participated in the competition she had aimed for and was able to deliver a satisfying performance. Five months post-treatment, she continues to compete without any recurrence of pain.

## Discussion

This case lacked any particular patient characteristics that would have promoted the development of nonunion at the fracture site. However, it is possible that the continuation of practice, even after the patient started experiencing foot pain and alternating between rest and practice, might have placed stress on the fracture site, impeding bone healing. By combining ESWT and PRP injections for the stress fracture of the proximal phalanx of the great toe, we achieved rapid bone union, which enabled a swift return to competition for the patient. While it has been reported that conservative treatment typically takes around two to three months for recovery, this treatment method shortened the time to bone healing and return to activity compared to conventional conservative treatments.

Focused ESWT on bone promotes the production of substances such as transforming growth factor-beta1, endothelial nitric oxide synthase, and vascular endothelial-derived growth factor, which in turn stimulates angiogenesis and bone formation [6-9]. It has been reported that focused shockwave therapy is effective in promoting the healing of delayed union fractures [10-12]. PRP is a plasma fraction obtained by centrifuging whole blood and contains a high concentration of platelets, which are rich in growth factors and anti-inflammatory cytokines. It is believed that PRP injections into bone might be effective for delayed union fractures or nonunions [13,14].

There has been only one report on the combined use of ESWT and PRP injection for fractures in the literature so far. Cen et al. reported on the utility of combined ESWT and PRP injection for nonunions. They compared the treatment outcomes of 60 cases with long bone nonunions classified into a PRP-only group and a combined ESWT and PRP group. The combined group showed significantly better results in terms of union rate and time to union. The combined use of ESWT and PRP was thought to have a synergistic effect on nonunion after fracture surgery [15]. The combination of ESWT and PRP injection may have potentially contributed to the healing of the delayed union in our case as well.

## Conclusions

In this case, the combination of ESWT and PRP injection for the stress fracture of the proximal phalanx of the great toe led to an exceptionally rapid bone union and return to competition. The combined use of ESWT and PRP injections, both beneficial for bone healing, may prove to be a potentially effective treatment for delayed union of the stress fracture of the proximal phalanx of the great toe.
